# Metabolomics reveals significant variations in metabolites and correlations regarding the maturation of walnuts (*Juglans regia* L.)

**DOI:** 10.1242/bio.017863

**Published:** 2016-05-23

**Authors:** Guodong Rao, Jinkai Sui, Jianguo Zhang

**Affiliations:** 1 State Key Laboratory of Tree Genetics and Breeding, Research Institute of Forestry, Chinese Academy of Forestry, Beijing 100091, Republic of China; 2 Collaborative Innovation Center of Sustainable Forestry in Southern China, Nanjing Forestry University, Nanjing 210037, Republic of China; 3 Key Laboratory of Tree Breeding and Cultivation, State Forestry Administration, Research Institute of Forestry, Chinese Academy of Forestry, Beijing 100091, Republic of China

**Keywords:** Metabolome, Walnut kernel, Developmental stages, ANOVA, Metabolite-metabolite correlation

## Abstract

The content of walnut metabolites is related to its nutritive value and physiological characteristics, however, comprehensive information concerning the metabolome of walnut kernels is limited. In this study we analyzed the metabolites of walnut kernels at five developmental stages from filling to ripening using GC-MS-based untargeted metabolomics; of a total 252 peaks identified, 85 metabolites were positively identified. Further statistical analysis revealed that these 85 metabolites covered different types of metabolism pathways. PCA scores revealed that the metabolic compositions of the embryo are different at each stage, while the metabolic composition of the endotesta could not be significantly separated into distinct groups. Additionally, 7225 metabolite-metabolite correlations were detected in walnut kernel by a Pearson correlation coefficient approach; during screening of the calculated correlations, 463 and 1047 were determined to be significant with r^2^≥0.49 and had a false discovery rate (FDR) ≤0.05 in endotesta and embryo, respectively. This work provides the first comprehensive metabolomic study of walnut kernels and reveals that most of the carbohydrate and protein-derived carbon was transferred into other compounds, such as fatty acids, during the maturation of walnuts, which may potentially provide the basis for further studies on walnut kernel metabolism.

## Abstract (precis)

**Summary:** This work provides a comprehensive metabolomic study of walnut kernels and reveals most of the carbohydrate and protein-derived carbons were transferred into other compounds during the maturation of walnuts.

## INTRODUCTION

Walnuts (*Juglans regia* L.) are classified as a strategic species and an indispensable food for human nutrition due to the high protein and oil content of its kernels(Martinez et al., 2010). Walnut trees, which are wildly distributed around the world, are cultivated commercially throughout southern Europe, northern Africa, the USA, western South America, and eastern Asia and have been characterized as an oleaginous tree for biodiesel fuels (Moser, 2012). Walnuts rank second behind almonds in tree nut production, and China leads the world production of walnuts according to the Food and Agriculture Organization (FAO). In 2010, global walnut production was 1,500,000 tons, with China accounting for 33.33% of the global walnut production (Mao and Hua, 2012). The walnut oil content of walnuts is 62-68%, thus making walnut oil the major product of the walnut; it is one of the most important specialty oils used for salad dressings and cooking due to the high content of monounsaturated and polyunsaturated fatty acids (Ros et al., 2004; Mao and Hua, 2012). Walnut protein is the main by-product of oil, which is considered to be another source of plant protein for use in human food products (Mao and Hua, 2014). Other contents, such as phenolic acids, tocopherols, flavonoids, juglone and phytosterols have also been researched (Martinez et al., 2010; Nour et al., 2013; Cosmulescu et al., 2014); it has been reported that the consumption of walnut kernel can provide protection against certain types of cancer due to its high concentration of natural antioxidants (Miraliakbari and Shahidi, 2008). Walnut nutrient composition has been investigated in several studies, the content of triterpenic, sterols, tocopherols, aliphatic alcohols, volatile compounds and carotenoids in the kernel oils from six walnut varieties were detected (Abdallah et al., 2015), and the level of tocopherols, fatty acids, total carotene and selenium of fifteen walnut varieties have also have been studied (Ozrenk et al., 2012). Molecular weight distribution, gel electrophoresis and amino acid composition of walnut proteins and protein fractionations were analyzed by high performance liquid chromatography (HPLC) to get a comprehensive understanding of the walnut proteins (Mao et al., 2014); however, a comprehensive study of the dynamic metabolite changes during walnut kernel ripening has not yet been published. Metabolomic analysis involves detecting and quantifying metabolic changes with techniques such as mass spectrometry and NMR spectroscopy, and integrating the resulting data with multivariate statistical techniques such as principal component analysis (PCA) and orthogonal signal correction projection to latent structure discriminant analysis (OPLS-DA) (Zhang et al., 2011;Topfer et al., 2015). In this study, a GC-MS based metabolomic approach was utilized to investigate the metabolic composition and natural metabolite variations during walnut kernel development. The measured metabolites were integrated into a metabolism network, and metabolite-metabolite correlation analysis was conducted. Results from this study will provide new insights into the understanding of walnut kernel metabolism, and create a more comprehensive overview of changes in metabolism during walnut kernel development and maturation.

## RESULTS

### Metabolomic profiling of ripening kernels in walnuts

**Fig. 1.**
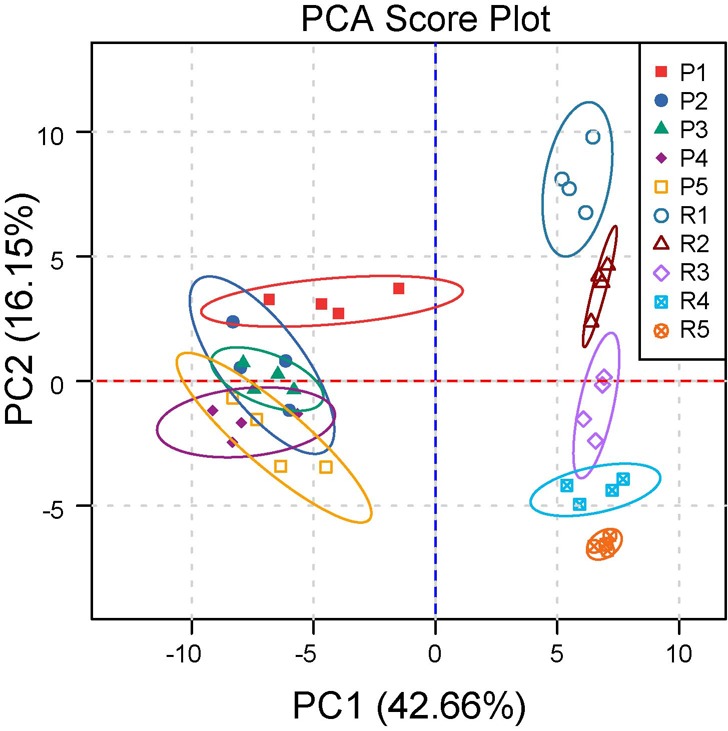
**Cluster analysis and PCA of metabolites in endotesta and embryos of walnuts.** PCA score plot generated from all 85 metabolites of different samples.

Kernels were sampled at five developmental stages from filling to ripening, with endotesta and embryos of kernels investigated separately. These developmental stages were identified as P1-P5 for endotesta, and R1-R5 for embryo. The metabolic profiling of kernels was investigated using an untargeted global metabolomics platform with GC-MS analysis. A total of 252 metabolites were detected, of which 85 named metabolites were confirmed using National Institute of Standards and Technology (NIST) and Wiley libraries. Hierarchical clustering analysis was performed to classify the walnut kernel metabolites, these 85 metabolites were classified into seven major metabolite groups which covered different metabolism pathways. The largest group contained 36.47% of the total number of identified metabolites: 31 organic acid metabolites. The second largest group had 20% of the total identified metabolites: 17 metabolites involved in carbohydrate metabolism. The third largest group (17.64%) consisted of 15 metabolites related to amino acid metabolism, followed by ten (11.76%) metabolites in amine metabolism, six (7.05%) in phosphate metabolism, and five (5.88%) in lipid metabolism. Principal component analysis (PCA) on all 85 metabolites was conducted to compare their metabolic composition throughout kernel development, with two principal components explaining 58.81% of the overall variance of the metabolite profiles, 42.66% and 16.15% for PC1 and PC2, respectively. The PCA scores revealed that metabolomes of the kernel and the endotesta are different from each other at all developmental stages (Fig. 1). The metabolic pathway of identified differentially abundant metabolites were then analyzed by the Kyoto Encyclopedia of Genes and Genomes (KEGG) which showed 63 identified metabolites that covered 14 pathways or metabolisms, including the citric acid cycle (TCA cycle), beta-alanine metabolism, D-arginine and D-ornithine metabolism, D-alanine metabolism, sphingolipid metabolism, propanoate metabolism, cysteine and methionine metabolism, pyruvate metabolism, arginine and proline metabolism, pyruvate metabolism, cysteine and methionine metabolism, pentose phosphate pathway, fructose and mannose metabolism, and glycolysis/gluconeogenesis (Fig. S2).

### One-way ANOVA analyses of the endotesta and embryo metabolites

**Fig. 2.**
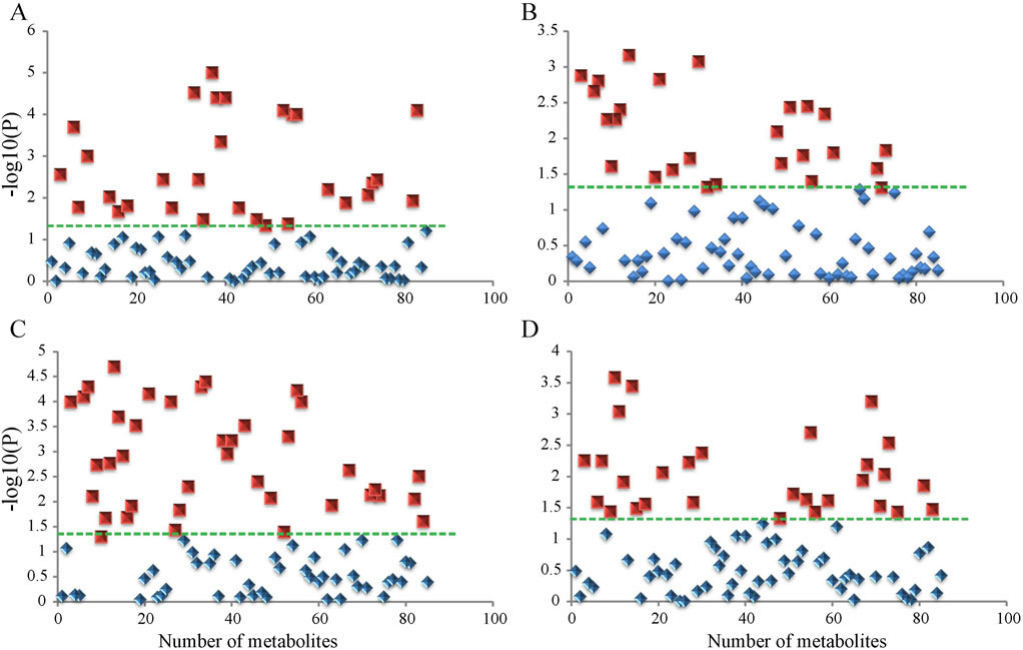
**One-way ANOVA analyses of endotesta and embryo metabolites in walnuts.** Metabolites identified as statistically significant (*P*<0.05; green dotted line) are shown as red squares while non-significant metabolites are shown as blue diamonds. (A) Metabolites of stage 1 to stage 5 in embryos. (B) Metabolites of stage 1 to stage 5 in endotesta. (C) Metabolites of stage 1, stage 3, and stage 5 in embryos. (D) Metabolites of stage 1, stage 3, and stage 5 in endotesta.

A one-way analysis of variance (ANOVA) statistical assay was performed to calculate the variations within the detected metabolites, *P*-values had been corrected for multiple hypothesis testing. The results revealed that levels of 30 metabolites in the embryos were differentially expressed at different stages of development (Fig. 2A), and levels of 26 metabolites of the endotesta were differentially expressed at the different stages of development (Fig. 2B). The 30 significantly differentially abundant metabolites from embryos included nine amino acids, eight organic acids, three amines and carbohydrates, and one phosphate. The 26 significantly differentially abundant metabolites from the endotesta contained eight amino acids, six organic acids, five carbohydrates, and three amines. We then calculated the variations of the metabolites in stage 1, stage 3, and stage 5 to further analyze the metabolite differences in bigger time span stages. The results showed that levels of 39 metabolites differed significantly in the embryo (Fig. 2C), and levels of 29 metabolites of the endotesta were significantly different (Fig. 2D). These significantly upregulated metabolites are mainly composed of amino acids and organic acids. Comparison analysis of significantly variable metabolites between all five stage and three stage metabolites revealed that the metabolites in the tissue of embryo are more variable compared to the tissue of teata, and the majority of these differentially abundant metabolites participate in amino acid and organic acid metabolism.

### Correlation analysis of endotesta and embryo metabolites in walnut

**Fig. 3.**
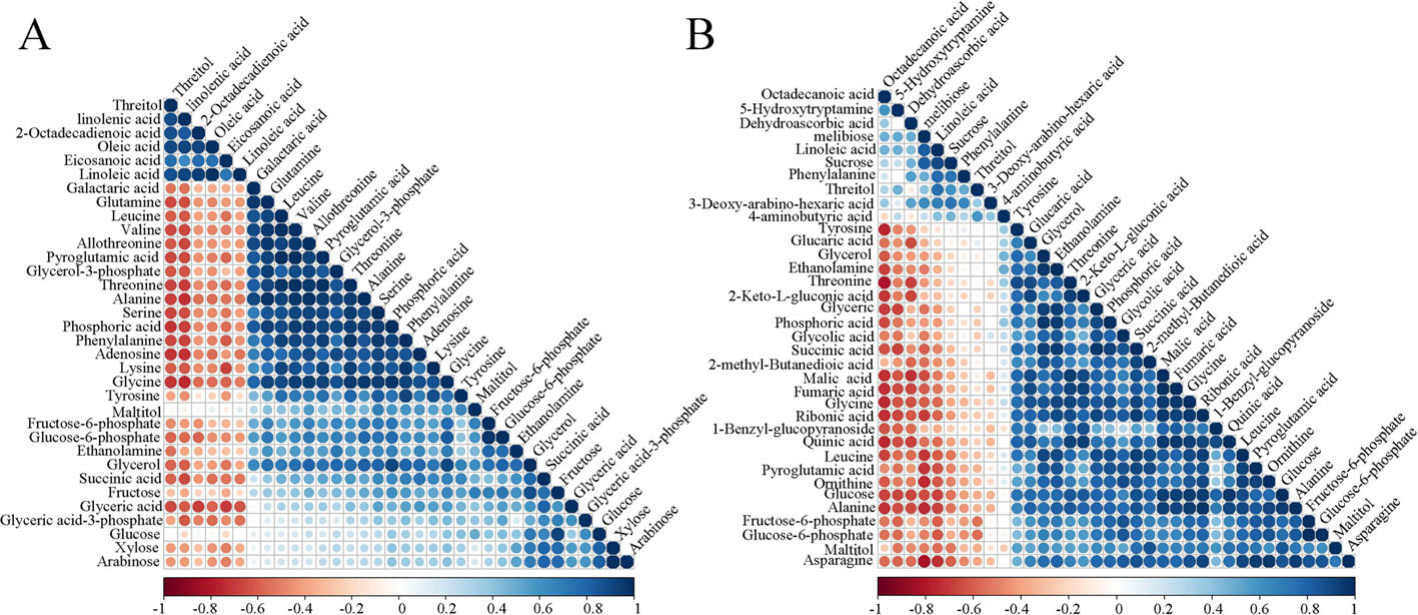
**Metabolite-metabolite correlation analysis.** Positive correlations are shown in blue; negative correlations are shown in red. (A) Metabolites-metabolites correlation of endotesta. (B) Metabolites-metabolites correlation of embryos.

Person correlation coefficient analysis was used to analyze the metabolite-metabolite correlation among identified metabolites in endotesta and embryo (Carrari et al., 2006; Blekhman et al., 2014). This analysis allowed for the identification of metabolites that related to each other in the two tissues across walnut development and ripening. Metabolite-metabolite correlations between the tissue of endotesta and embryo showed unique profiles; in endotesta 1156 resulted in significant correlation coefficients (*P*<0.01), out of these 1156 significant correlations, 790 were positive and 366 were negative (Fig. 3A). Notably amino acids dominated the significant metabolite correlations, followed by organic acids. In embryo, 1225 resulted in significant correlation coefficients (*P*<0.01), out of these 1225 significant correlations, 715 were positive and 510 negative (Fig. 3B), amino acids and organic acids also dominated the significant metabolite correlations.

Further screening of the calculated correlations was performed, and 463 were determined to be significant with r^2^≥0.49 and had a false discovery rate (FDR) ≤0.05 in endotesta, among which 258 were positive correlations and 205 were negative correlations (Fig. S3). In the 258 positive correlations, organic acids dominated the most significant metabolite-metabolite correlations, followed by amino acids, carbohydrates, and amines. In embryos, there were 1047 significant correlations with r^2^≥0.49 and FDR ≤0.05 identified; among them, 911 were positive correlations while 136 were negative correlations. Organic acids and amino acids also played important roles in metabolite correlations.

## DISCUSSION

Kernel maturity is an important developmental stage of plant reproduction, and the metabolites in kernels relate to kernel quality and nutritional values. Walnut is an agronomically important tree with high nutritional value due to the high contents of oils, proteins, and many other beneficial metabolites in the kernel. In this study, differential quantitative metabolite profiling was used to assess developmental changes of walnut kernels. Endotesta have the main function of protection of the embryo and also have a linkage between embryo and outer environment. The levels of metabolites in endotesta were relatively stable during walnut kernel maturation, PCA scores also revealed the metabolic compositions in this tissue could not be separated. Embryos are the energy source of kernels, metabolites in this tissue are synthesized for the germination as seed. The levels of most metabolic compositions in embryo were increased during the development of kernels. PCA scores also proved metabolites of the embryo are different at each stage of development.

**Fig. 4.**
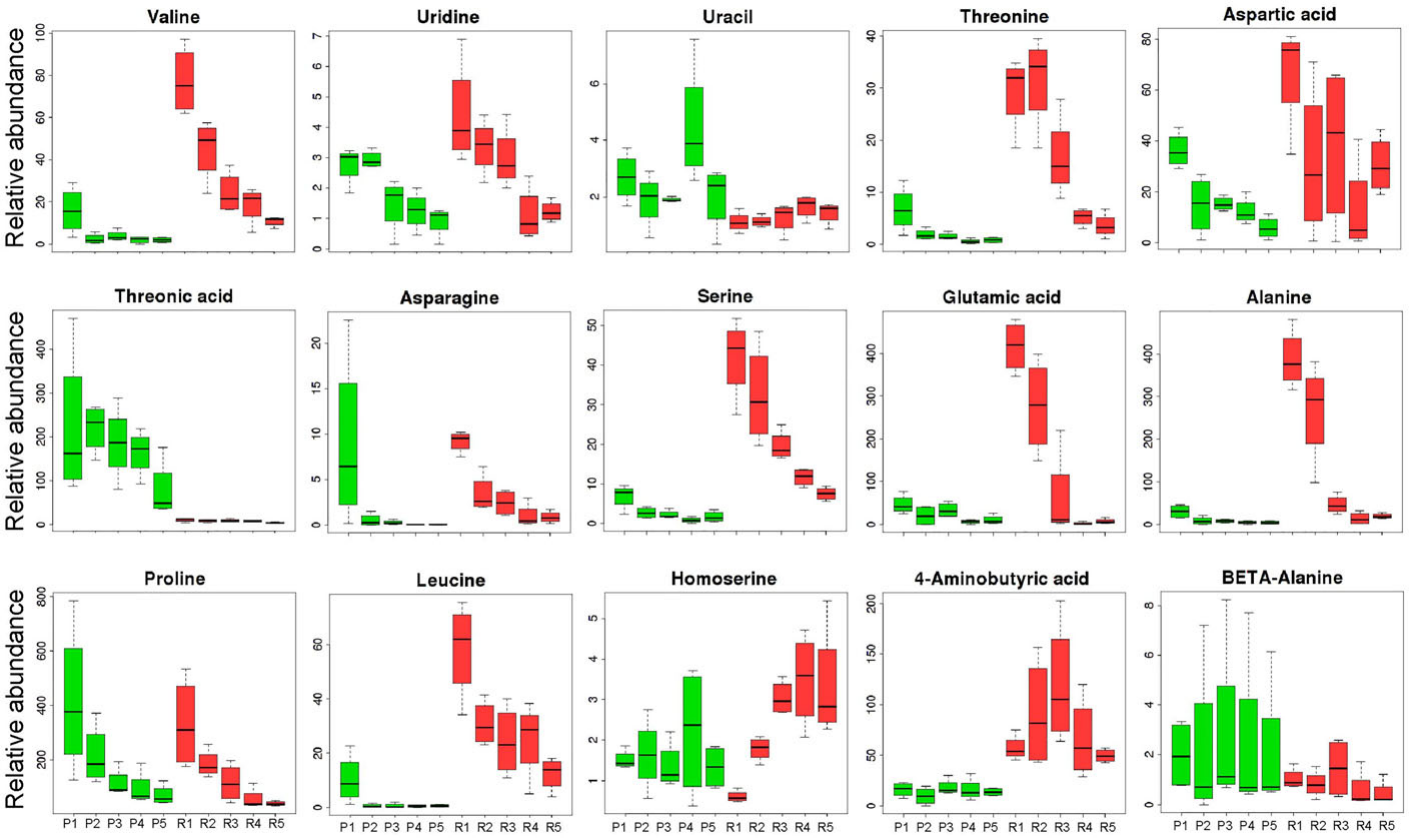
**Boxplot-visualizations of amino acid relative abundances in endotesta and embryo of walnut kernels.** The relative abundance of endotesta amino acids at different time points (1-5) are show in green, and the relative abundance of embryo amino acids at different time points (P1-P5; R1-R5) are show in red.

**Fig. 5.**
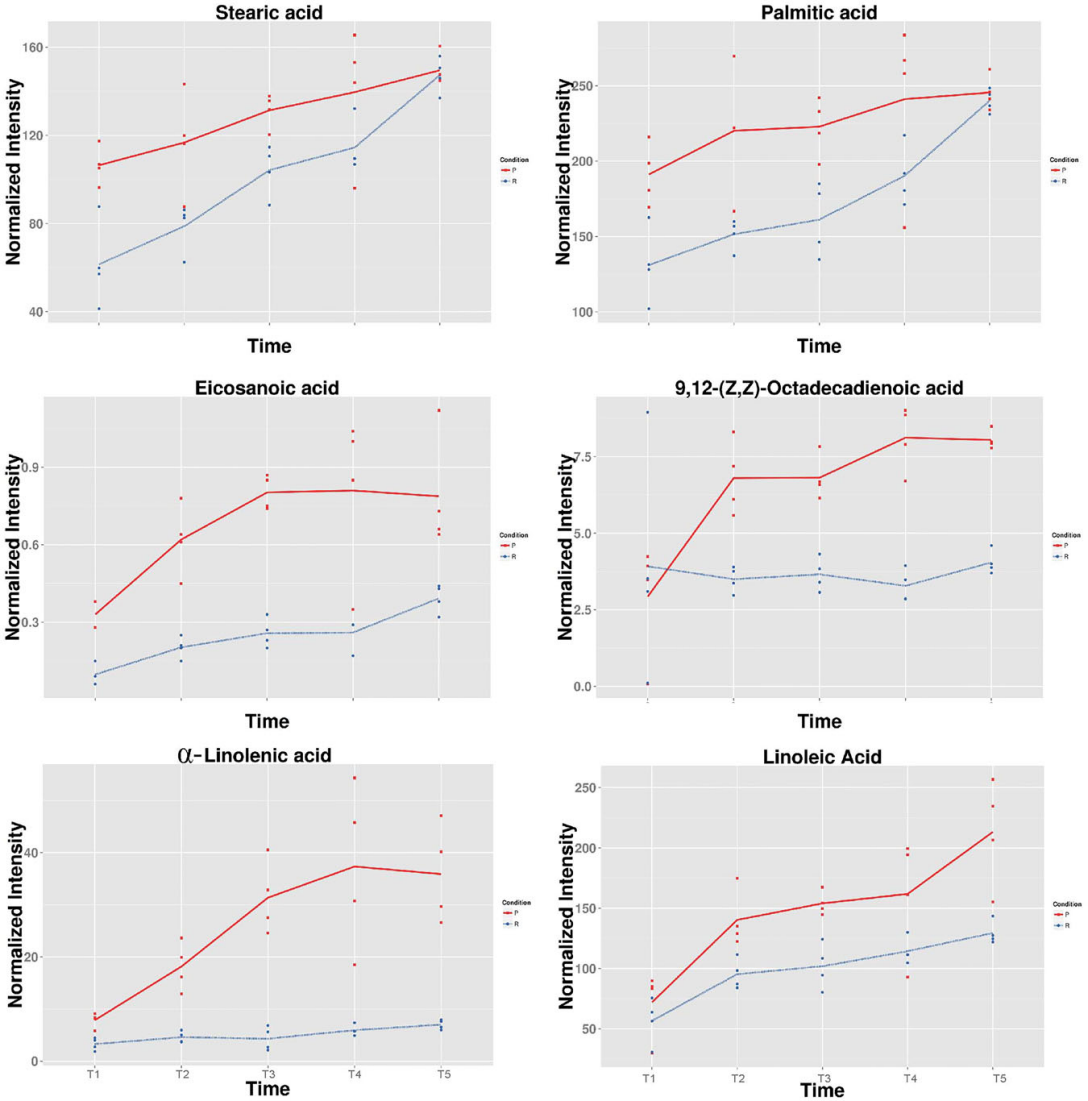
**Fatty acids normalized intensity in endotesta and embryo of walnut kernel.** The normalized intensity of endotesta organic acids (P) at different time points (T1-T5) are show in red, and the normalized intensity of embryo amino acids (R) at different time points are show in blue.

Levels of most amino acids were stable, however, not for asparagine, proline, and leucine in endotesta, which decreased during kernel ripening (Fig. 4). Asparagine is a nitrogen transport compound found in many plants, it accounts for 50-70% of the nitrogen carried in translocatory channels serving fruit and seed of white lupin (*Lupinus albus* L.) (Atkins et al., 1975). In soybean, asparagine was proven to have a positive correlation with protein concentration in developing seeds, and the level of asparagine synthesis was high initially and progressively declined in the seed coat (Pandurangan et al., 2012). In the present study the level of asparagine was over tenfold higher in the early stage (stage 1), suggesting that the development of kernels requires a large amount of nitrogen which can be used for protein synthesis at the early stage. Many studies have shown that proline plays a regulatory role in response to biotic and abiotic stress by increasing its content. A functional link between elevated proline and the ability of tolerance stress of seeds has been demonstrated in *Arabidopsis thaliana* (Hare et al., 2003). In sorghum seeds, proline also has the ability to increase the antioxidant compounds and oil antioxidant activity under normal and drought stress conditions in maize seed (*Zea mays* L.) (Ali et al., 2013). The level of proline is much higher at the early stage compared to other stages and progressively declines from stage 1 to stage 5, suggesting high levels of proline are required at the beginning of kernel development for preventing stress-induced damage. Observations have shown that leucine-derived carbon is incorporated into sugars and organic acids and is an alternate source of acetyl-CoA to sustain respiration and metabolic processes (Anderson et al., 1998). In the present study, leucine is present only at the early stage (stage 1), and barely detected at the later stages (stages 2-5), suggesting that leucine at stage 1 provides leucine-derived carbon for the subsequent synthesis of other compounds (sugars or organic acids) at the following stages. Endotesta are the barrier between the embryo and outer environment, and proteins in this tissue are mainly comprised of structural proteins, regulatory proteins, and transport proteins. The levels of these proteins are stable during the kernel ripening, which complies with the stability of amino acid levels noted in comparison with the embryo. The embryo is the main part of seed, consisting of precursor tissues for leaves, stem and root. Proteins in the embryo mainly comprise of storage proteins which provide the energy for kernel germination. High levels of amino acids were detected at the early stage and the level of most amino acids decreased during the development of kernel in embryo until only low levels were detected at stage 5 (Fig. 5), suggesting that most of the protein-derived carbon was transferred into other compounds during kernel development. A targeted global metabolomics platform with GC-MS was used to verify the accurate content of amino acids of kernels, which showed the same variation of trends of amino acids contents detected by an untargeted method. A total of 20 amino acids with a decreasing pattern across five development stages were identified, the total content of amino acids decreased from 3282.65 µg/g at T1 to 369.80 µg/g at T5 (Table 1).

**Table 1.**
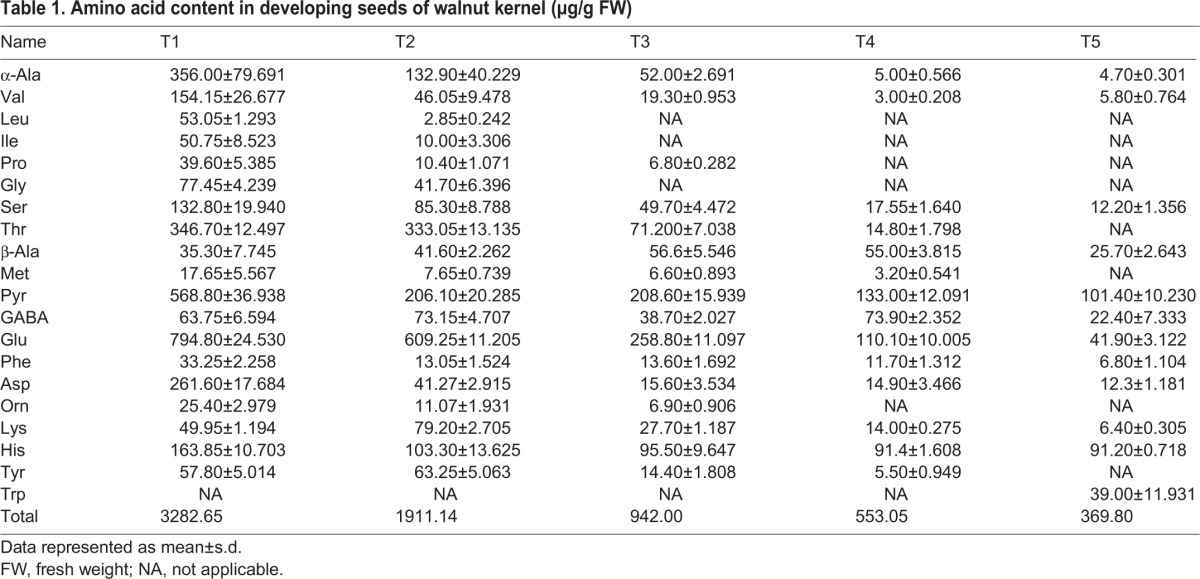
**Amino acid content in developing seeds of walnut kernel (µg/g** **FW)**

Fatty acids are the main component of plant seed oil and are usually unbranched compounds with an even number of carbons ranging from 12 to 22 and with 0 to 3 cis double bonds (Thelen and Ohlrogge, 2002). Six fatty acids including stearic acid, palmitic acid, α-linolenic acid, eicosanoic acid, 9,12-(Z, Z)-octadecadienoic acid, and linoleic acid were detected by the untargeted metabolomic during walnut kernel development. A targeted global metabolomics platform with GC-MS was used to verify the accurate content of fatty acids of kernels. A total of twelve fatty acids with an increasing pattern across five development stages were identified. The total content of fatty acids were accumulated from 15.02 mg/g at T1 to 157.58 mg/g at T5, among which the unsaturated fatty acids were accumulated from 10.81 mg/g at T1 to 127.24 mg/g at T5 (Table 2).

**Table 2.**
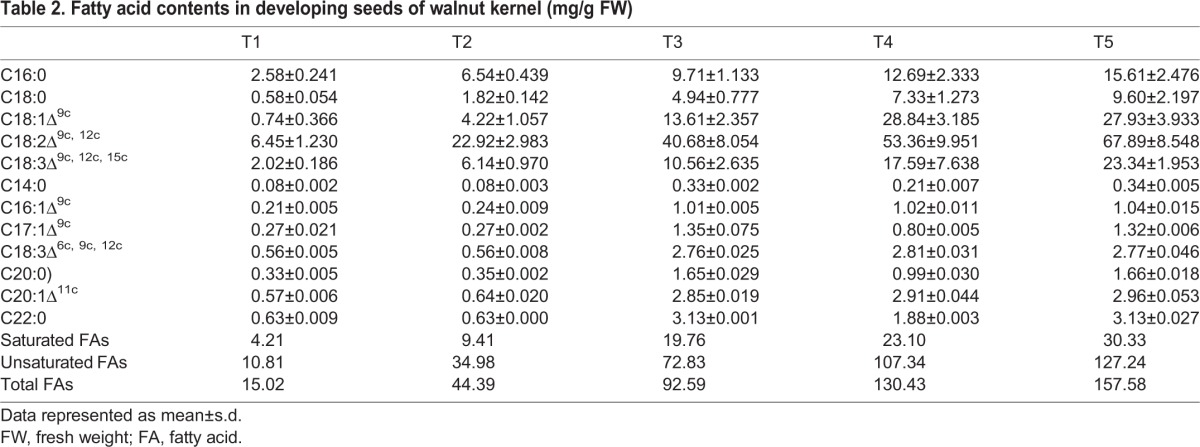
**Fatty acid contents in developing seeds of walnut kernel (mg/g** **FW)**

The level of these six fatty acids increases during the kernel development (Fig. 5), indicating a continuous carbon flow into fatty acids from other compound such as amino acids mentioned above. Unsaturated fatty acid content is one of the important parameters of different vegetable oils. Linoleic acid and α-linolenic acid are the predominant unsaturated fatty acids found in most seed oils, which are required in the diet of higher animals, including humans, as they lack the desaturase that convert oleic acid to linoleic acid and α-linolenic (Gong et al., 2014). Fatty acids also have the function for plant tolerance biotic and abiotic stresses by affect the fluidity of cell membrane (Upchurch, 2008). Linoleic acid is the most abundant fatty acid in plant membranes, the increasing accumulation during the kernel ripening suggesting it may be involved in modifying membranes fluidity necessary for the development of walnut kernel. Free α-linolenic acid has been proved to possess antifungal activity in many plants (Göbel et al., 2002; Walters et al., 2004; Sánchez-Sampedro et al., 2007); it is also a precursor of jasmonic acid which plays a vital role in plant responses to biotic and abiotic stress (Wasternack, 2007; Wasternack and Hause, 2013). The accumulation of α-linolenic acid provided an indicator for the walnut fungal defense response during the kernel development. Three saturated fatty acids (stearic acid, palmitic acid, and eicosanoic acid) were detected to have increased levels during kernel ripening. The level of stearic acid and palmitic acid are much higher than eicosanoic acid, indicating stearic acid and palmitic acid are the main saturated fatty acids in walnut kernel. Interestingly, the levels of stearic acid and palmitic acid are different at the early stage of kernel development, while these two saturated fatty acids continuously increase to the same level until ripening, suggesting not only that the embryo has high levels of fatty acids but high levels of fatty acids were in endotesta.

**Fig. 6.**
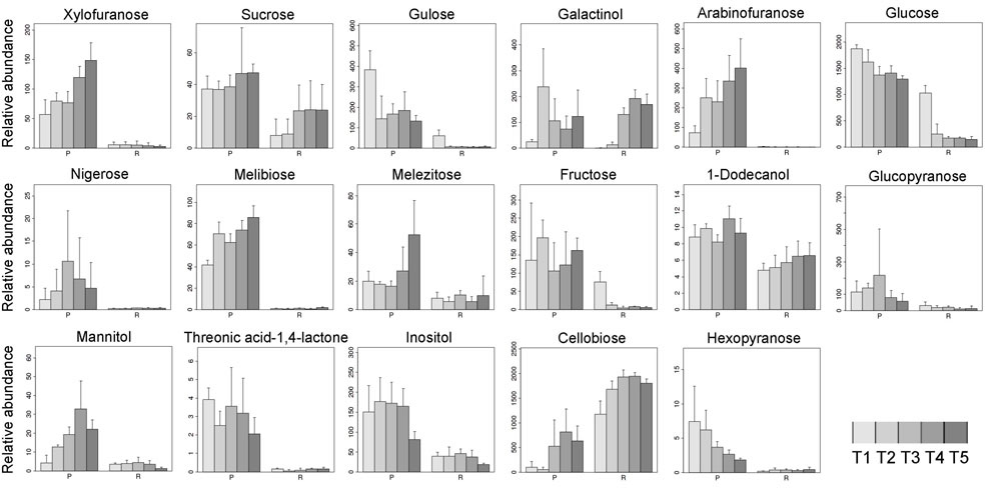
**Relative abundance of carbohydrates in endotesta and embryo of walnut kernels.** Bar graph shows the normalized abundance of carbohydrates in endotesta (P) and embryo (R) at different development time points (T1-T5). Data represented as mean±s.d.

The levels of carbohydrates were higher and relatively stable in endotesta, while they were more dynamic during the kernel development in embryo (Fig. 6). Glucose and cellobiose were predominant during the walnut kernel development; the difference between these two carbohydrates was that the level of glucose was continuously decreasing while cellobiose increased to a high and stable level during kernel ripening. The reverse content changing between these two carbohydrates supports the carbon flow into cellobiose from glucose. Glucose is an energy source that plays an important role in the respiration of several plant tissues. In endotesta the levels of glucose were stable during development, suggesting that there is a balance between the glucose consumed and synthesized. Meanwhile, the glucose levels at the early stage were much higher than in the later stages in the embryos, suggesting that the glucose was mainly consumed as an energy source for respiration and metabolic synthesis. Cellobiose can be obtained by enzymatic or acidic hydrolysis of cellulose and cellulose rich materials, suggesting that the main carbohydrate of the ripe walnut kernel is cellulose. In higher plants, cell morphogenesis and anisotropic growth were mediated by a cellulose biosynthesis process which played a vital role to support the structural framework of the plant cell wall (Mendu et al., 2011). Cellulose also has been considered to have the key role to strengthen the secondary cell wall, which protects the embryo during seed development (Stork et al., 2010). Cellulose is also protects the seed from damage in the environment by seed mucilage that surrounds the seed (Arsovski et al., 2010). The dynamic upregulation of cellulose both in endotesta and embryo of walnut kernel suggests that cellulose plays an important role in carbohydrate metabolism during kernel development. Another two carbohydrates, gulose and fructose, have the same dynamic changing model as glucose; both of them were consumed at the early stage from a relative high level in embryos, indicating that these two carbohydrates are essential for the development of embryos at the early stage either by providing energy sources or as a chemical structure of enzymes.

## MATERIALS AND METHODS

### Plant materials

Four representative walnut (*Juglans regia* L.) cultivar kernels at five developmental stages were obtained from the Chinese Academy of Forestry, and the endotesta and embryos of kernels were collected separately. The five developmental stages span from fruit filling to ripening which were harvested at 15 (stage 1), 30 (stage 2), 45 (stage 3), 60 (stage 4), and 80 (stage 5) days after full bloom (DAF). Embryos were aquiform at early stages (stage 1 and stage 2) and hardened as time went on (stage 3 to stage 5). All samples were frozen in liquid nitrogen immediately and stored at −80°C until further processing.

This study was carried out in strict accordance with the recommendations in the guide for observational and field studies (http://bio.biologists.org/content/manuscript-prep). All necessary permits were obtained for the described field studies. The sampling of all individuals of *J. regia* L. were approved by Shougong Zhang, director of Chinese Academy of Forestry.

### Metabolite extraction, derivatization for GC-MS

Samples were ground under liquid nitrogen to obtain a fine powder, and 100 mg of lyophilized powder per sample was weighed for metabolite extraction. Cold extraction was employed, and the extraction protocol was followed according to previous studies with slight modifications (Weckwerth et al., 2004). 1 ml of cold solvent (maintained at −20°C), comprised of methanol:chloroform:water in a ratio of 5:2:1 together with 50 µl of internal standard (Ribitol stock concentration, 0.2 mg/ml), was added to the ground material and vortexed for 10 s. The mixtures were then kept on ice for 25 min with intermittent vortexing or shaking for 10 s. The homogenate was then centrifuged at 13,000×***g***, at 4°C for 10 min and the supernatant was transferred to a new tube with 200 µl chloroform and 500 µl of deionized water and vortexed again. The mixture was then centrifuged at 13,000× ***g***, at 4°C for 5 min. The solution was separated into the upper and lower phases; the upper phase was aqueous and the lower phase was organic. The aqueous phase was then dried under vacuum and derivatized for GC-MS analysis.

Derivatization steps were carried out as follows. The dried organic phase was dissolved in 20 µl of methoxyamine hydrochloride (M. HCl) solution (40 mg/ml in pyridine) and was kept at room temperature for 2 h. Subsequently, 80 µl of N-methyl-N-(trimethylsilyl) trifluoroacetamide (MSTFA) was added to the M. HCl mixture and incubated at 37°C for 30 min with shaking. The derivatized samples were centrifuged at 13,000× ***g*** for 5 min and then transferred to autosampler vials for GC-MS analysis. A mixed n-alkane standard solution C8–C20 and C21–C40 (Sigma Aldrich, St Louis, MO, USA) was used for the determination of retention indices (RI). The sample set also included a quality control (QC) with an aliquot (40 µl) of all prepared sample extracts mixed.

### Untargeted metabolomic analysis

The derivatized samples were analyzed using a global unbiased mass spectrometry-based platform with GC-MS, and data normalization was performed according to a previous study (Zhang et al., 2011). The samples were randomized and the data acquisition was done in one batch to eliminate system errors. GC-MS was carried out on an Agilent 7890A/5975C GC-MS and auto-sampler unit. An HP-5MS (Agilent J&W Scientific, Folsom, CA) column with 0.25 µm thickness, 250 µm diameter, and 30 m length was used to separate derivatized metabolites. 1 µl of sample was injected in split mode in a 1:20 split ratio by the auto-sampler. The injection temperature was set at 280°C and the column oven temperature was 80°C, with helium as the carrier gas. Mass spectrometry settings were as follows: ion source temperature was 250°C, interface temperature was 280°C, and solvent cut time was 5 min. Temperature program: 5 min hold at 40°C, followed by 10°C/min ramp to reach a final temperature of 300°C held for 5 min. The scan range was 35-750 m/z.

Raw GC/MS data were converted into CDF format (NetCDF) files by Agilent GC/MS 5975 Data Analysis software and subsequently processed by the XCMS (www.bioconductor.org) using XCMS's default settings with the following changes: xcmsSet (fwhm=3, snthresh=3, mzdiff=0.5, step=0.1, steps=2, max=300), group (bw=2, minfrac=0.3, max=300). The signal integration area of each metabolite was normalized to the internal standard (ribitol) for each sample. Identification of metabolites using the automated mass spectral deconvolution and identification system (AMIDS) was searched against commercial available databases such as National Institute of Standards and Technology (NIST) and Wiley libraries. Metabolites were confirmed by comparison of mass spectra to the spectra library using a cut-off value of 70%, and by matching the experimental retention time index (RI) of each metabolite with the mass spectral and RI collection of the Golm Metabolome Database (GMD). Principle component analysis (PCA) was performed with R software (www.r-project.org). Heat map packages available in R were used to draw heat maps, and Mev (MultiExperiment Viewer) 4.8 software was used to perform a one-way ANOVA with standard Bonferroni correction. Identified metabolites were mapped onto general biochemical pathways according to annotation in KEGG. Metabolic network maps were constructed by incorporating the identified and annotated metabolites using Cytoscape 3.2.0 software (http://www.cytoscape.org/).

### Targeted metabolomic analysis of fatty acid and amino acid

The extraction of fatty acids was conducted with a chloroform-methanol (1:2) mixture, according to the method of previous study (Bligh and Dyer, 1959; Thurnhofer and Vetter, 2005). Fatty acids were transesterified with 2 ml 1% methanol with an internal standard (D-25 tridecanoic acid) were added in sulfuric acid for 2 h at 80°C, then 2 ml μl H_2_O and 2 ml hexane were added and vortex mixed. The derivatised organic layer was injected in an Agilent 7890A/5975C GC-MS in the selected ion monitoring (SIM) mode with a DB-WAX (Agilent J&W Scientific; 30 m×0.25 mm ID×0.25 μm) column. The initial column temperature was 50°C, after held for 3 min the column temperature was increased to 220°C by 10°C per minute, and held for 20 min. The MS ion source temperature was 230°C and the Quadrupole temperature was 150°C. Supelco 37 component FAME mix (Sigma-Aldrich) were used as reference standards to identify the fatty acids. The quantity of each methyl fatty esters was calculated from calibration curves of standards. For analysis of soluble amino acid content, 50 mg of samples were ground to powder and extracted in 1 ml methanol:chloroform:water in a ratio of 5:2:1 together with Norvaline (50 µl 50 µg/ml) as an internal standard and selected ion monitoring (SIM) mode was performed instead of full scan mode (Amira et al., 2005). The quantity of each amino acid was calculated from calibration curves of standards.
